# What's new in IL-12? Combination!

**DOI:** 10.1186/2051-1426-3-S2-P363

**Published:** 2015-11-04

**Authors:** Chris E Lawrence, Mark Rubinstein, Zoya Gluzman-Poltorak, Vladimir Vainstein, Lena A Basile

**Affiliations:** 1 Neumedicines, Pasadena, CA, USA; 2 Medical University of South Carolina, Charleston, SC, USA

## 

IL-12 is a heterodimeric, pro-inflammatory cytokine that enhances the cytotoxic activity of natural killer (NK) cells and cytotoxic CD8^+^ T-lymphocytes, and induces an IFN-α-dominated Th1 CD4^+^ T-lymphocyte response. IL-12 as an immunotherapeutic agent administered subcutaneously in cancer patients has demonstrated clinical responses in melanoma, T cell lymphoma, non-Hodgkin's lymphoma, and AIDS-related Kaposi sarcoma, but was never developed further. Having elucidated novel hematological properties of IL-12, we are advancing our proprietary recombinant human IL-12 (NM-IL-12) for the treatment of the Hematopoietic Syndrome of Acute Radiation Syndrome. In three clinical safety studies conducted in over 200 healthy human volunteers, subcutaneous NM-IL-12 was well-tolerated. No adverse immune reactions or immunogenicity were observed.

We have now developed a novel clinical paradigm for the use of subcutaneously administered, low dose NM-IL-12 combined with standard of care radiotherapy, chemotherapy, or immunotherapy for the treatment of cancer. The pleiotropic effects of IL-12 are expected to augment the mechanistic, anti-tumor effects of each of these treatments.

*In vitro* NM-IL-12 stimulated primary human NK cell secretion of IFN-g and the cytotoxic lysis of leukemic cells, and inhibited production of pro-angiogenic IL-17 in human peripheral blood mononuclear cells. *In vivo*, recombinant murine IL-12 (rMuIL-12) caused significant tumor growth inhibition following total body irradiation (625cGy) in syngeneic Lewis lung and EL4 lymphoma tumor models. In the same models, rMuIL-12 in combination with cyclophosphamide also caused significant tumor growth inhibition. In the case of the non-immunogenic Lewis lung cancer model the combination of chemotherapy and IL-12 enhanced immunogenicity. In both tumor models, the antitumor effects of IL-12 were accompanied by rapid recovery of neutrophils, platelets and red blood cells, depressed by radiation or chemotherapy. This suggests an additional benefit of NM-IL-12 to cancer patients myelosuppressed following radiation or chemotherapy.

Preclinical evaluation of NM-IL-12 with radiation therapy and chemotherapy is now followed by evaluating combination immunotherapy. PD-1 blockade elicits potent anti-tumor immunity in a subset of melanoma patients. We have thus evaluated the combination of rMuIL-12 and anti-PD-1 antibody in a clinically relevant, syngeneic model of spontaneous, highly metastatic B16 mouse melanoma has been tested.

In summary we show that NM-IL-12 has excellent anti-tumor potential when used preclinically in combination with standard of care anti-cancer treatments, including radiation, chemotherapy and immunotherapy. NM-IL-12 is expected to contribute durable anti-tumor responses in the clinic through potent immunoactivation and anti-angiogenic effects, and to replenish blood cells, while being safe, well tolerated and non-immunogenic.

